# Countries’ positions in the international global value networks: Centrality and economic performance

**DOI:** 10.1007/s41109-017-0041-4

**Published:** 2017-07-12

**Authors:** Isabella Cingolani, Pietro Panzarasa, Lucia Tajoli

**Affiliations:** 1 Big Data and Analytical Unit, Department of Surgery and Cancer, Imperial College London, London, UK; 2 School of Business and Management, Queen Mary University of London, London, UK; 3 Department of Management, Economics and Industrial Engineering, Politecnico di Milano, Milan, Italy

**Keywords:** Global value networks, Centrality, Market power, Upstreamness, Midstreamness, Downstreamness

## Abstract

The international exchange of goods and services is increasingly organised along global value chains in which the various production stages are carried out at many different locations all over the world. A country can be seen as holding a central position in global trade to the extent that it is involved in a large number of economic transactions with alternative potential suppliers and has a wide access to different important markets. However, the centrality of countries’ positions in the international production of goods and services may vary according to the specific stages of the production process that countries occupy. Here we adopt a network-based perspective, and propose a novel three-faceted measure of centrality that captures countries’ distinct roles at the upstream, midstream, and downstream stages of the international production process. Findings suggest that rankings of countries based on our measures of centrality vary across production stages. While emerging and developing countries tend to secure central positions at upstream and midstream production stages, high-income countries tend to exert prevailing roles at downstream stages. Moreover, rankings based on our measures differ from alternative rankings obtained from traditional measures of market power simply reflecting aggregate trade values. This is especially the case within more traditional industries, such as Textiles and Apparel, in which small and less developed countries can play relevant roles at various stages of the production process.

**Electronic supplementary material:**

The online version of this article (doi:10.1007/s41109-017-0041-4) contains supplementary material, which is available to authorized users.

## Introduction

The increasing relevance of global value chains (GVCs) in international trade has been widely emphasised by a number of recent studies (e.g., Baldwin and Lopez-Gonzales 2015; Johnson and Noguera 2012; Timmer et al. 2014). GVCs are the result of production processes stretching across multiple countries (i.e., the international fragmentation of production), so that different phases of the production of a final good are undertaken in different countries to exploit the specific comparative advantage of each location. Such international organisation of production is responsible for the occurrence of multiple trade exchanges among countries, which have recently been estimated to represent about half of all trade flows (Baldwin and Lopez-Gonzales 2015).

In this work, we draw on data concerned with world trade in three different sectors to uncover the network structure and dynamics of GVCs. It is reasonable to expect that the organisation of trade flows driven by international production linkages differs from the one of aggregate traditional trade flows, especially in sectors, like the ones examined here, where the internationalisation of production processes is widespread. Trade flows related to intermediate inputs connecting production phases do not necessarily mirror the trade flows originating from the main producers of a given product and directed toward the largest markets of consumers. Rather, GVCs might involve intermediaries and assembly spots located in countries far away from the technological frontier or in countries holding only a small share of the international market of the final product.

This has prompted our interest in assessing the centrality of countries in the global network of links generated by international production processes. Indeed, according to extant economic models, the position of a country within an international production system is correlated with the share of value it adds to production and with the share of benefits it obtains from trade (Costinot et al. 2013; Johnson and Noguera 2012). To shed light on a country’s participation in global trade, it is therefore important to understand where the country is positioned in a GVC, and how relevant its role is at the various stages of production. There is a growing literature concerned with measures for assessing a country’s participation and position in GVCs (e.g., Antràs and Chor 2013; Antràs et al. 2012; Daudin et al. 2011; Koopman et al. 2014; Wang et al. 2013), and our work aims to contribute precisely to this stream of literature by adopting a network analysis approach to international production and trade.

An advantage of this approach lies in the fact that it does not assume international trade to occur merely from producers to final consumers. Rather, it allows us to describe international trade more appropriately as an international production network arising from a sequence of production steps involving potentially multiple countries before the final product can reach the final destination markets of consumers. Indeed countries in such production network may receive inputs from multiple sources and may contribute to the production process at multiple stages located in more than one country. To fully assess the role that countries play in international trade, it is therefore crucial to distinguish between flows of intermediate products and flows of final ones, and then uncover the position countries occupy at each production step of the underlying GVC.

We begin by assessing the salience and distinctive properties of the network measure of centrality within the broad context of the international global value networks (GVNs). We then describe the bilateral trade data set on which our study draws, and outline the procedure used to distinguish between intermediate and final products and to extract the international GVNs from the underlying international aggregate trade networks. Based on a tripartite valued and directed graph, we propose a formalisation of a three-faceted measure of centrality that captures the distinct roles each country plays as an exporter or importer at the upstream, midstream, and downstream stages of the production process. We evaluate our centrality measures in three industries (Electronics, Motor Vehicles, and Textiles and Apparel), and assess their evolution between 2007 and 2014. We then compare rankings of countries based on our centrality measures with alternative rankings based on traditional economic measures based on gross value of exports and imports. We conclude by summarising and discussing our results, outlining their limitations, and suggesting avenues for future work.

## Centrality and the global value network

Centrality has long been a fundamental concept in the study of various types of networks. Most measures of centrality so far advocated aim to capture a node’s structural importance by computing its involvement in the walk structure of a network (Borgatti and Everett 2006; Freeman 1978). Among various empirical applications, centrality has been used to investigate power (Brass 1984; Burt 1982), social influence in inter-organisational networks (Laumann and Pappi 1973; Galaskiewicz 1979), adoption and diffusion of innovation (Coleman et al. 1966), and employment opportunities (Granovetter 1974).

While centrality measures have mostly been developed for one-mode, binary, and time-invariant networks, there have been a number of attempts to extend such measures to two-mode networks (Faust 1997), or more generally *K*-partite graphs, as well as weighted (Barrat et al. 2004), multiplex (Boccaletti et al. 2014; Menichetti et al. 2014; Rahmede et al. 2017) and time-varying networks (Nicosia et al. 2013). Attempts have also been made to cross-classify centrality measures in terms of various criteria, including their underlying assumptions on how processes unfold in a network (e.g., the trajectories followed and the method of spread) (Borgatti 2005), and the type of nodal involvement and property of walk assessed (Borgatti and Everett 2006).

Recently, a number of studies have drawn upon the network literature to develop suitable sets of metrics to capture the role that countries play in the international GVCs. For instance, it has been suggested that measures for identifying hubs and spokes at the sectoral level can help uncover the connection between global trade patterns and global supply chains (Lejour et al. 2014). Yet, what is still largely missing is an analytical framework in which the centrality of a country is explicitly formalised as a multi-faceted measure that captures the country’s position at the various production stages into which the international GVNs are organised.

The international GVN in a given industry can be regarded as a special case of *K*-partite weighted and time-varying networks in which goods and services flow from one country to another following complex trajectories along the production process, typically ending in final consumption. These trajectories can be seen as organised into *K* distinct stages that can be traversed by goods and services along one distinct direction – i.e., from upstream toward downstream – yet through transactions in which the same country can occupy multiple roles. For example, country *n*_*i*_ may export an intermediate product to country *n*_*j*_ to be transformed, and then import from the same country *n*_*j*_ the output of such transformation that, in turn, will be further transformed. Thus, the flow process of production may involve the same country multiple times. Moreover, links between countries are weighted as, by construction, they are associated with the volume or value of the goods and services exchanged. Finally, as the production process unfolds over time, each transaction is time-stamped and the underlying network is therefore time-varying.

**Fig. 1:**
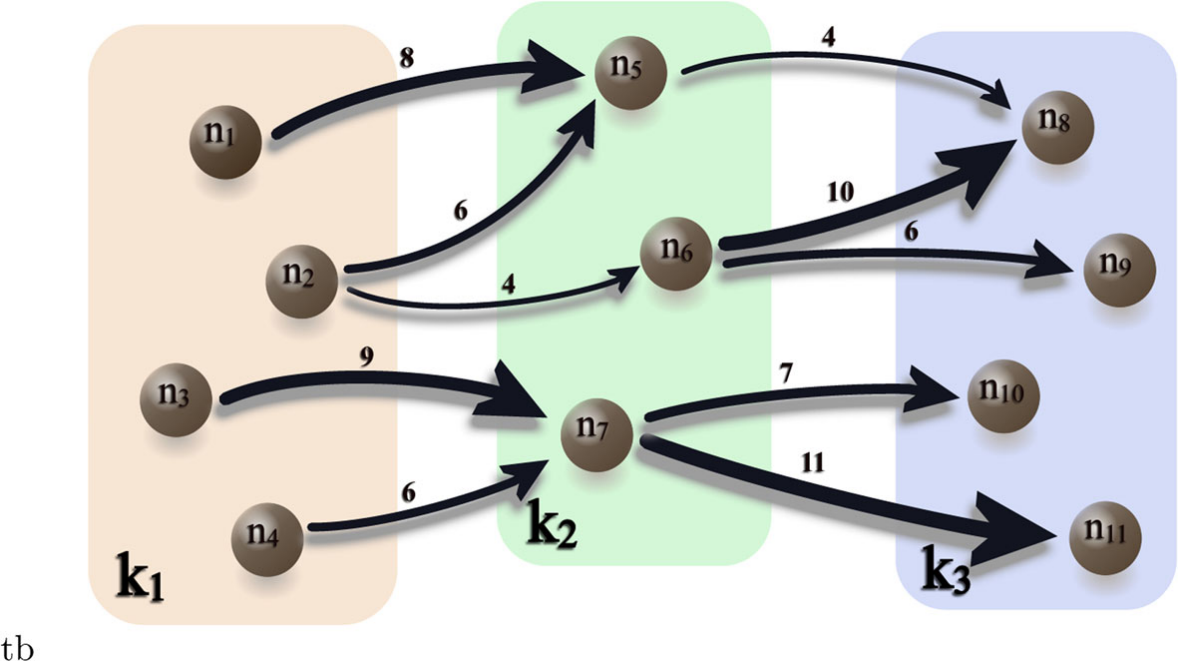
Example of an input-output tripartite weighted graph. The sets *k*_1_, *k*_2_, and *k*_3_ represent three production stages. Only links from nodes in set *k*_1_ to nodes in set *k*_2_ and links from nodes in set *k*_2_ to nodes in set *k*_3_ are allowed. Above each link is indicated the value of its corresponding weight. The width of each link is proportional to the corresponding weight

Formally, a GVN is an input-output *K*-partite graph with *K* sets of nodes such that nodes in each set *k*_*i*_ are only linked to nodes in set *k*_*i*+1_ for *i*<*K*. Thus, nodes in set *k*_1_ only send links to nodes in set *k*_2_, nodes in set *k*_*K*_ only receive links from nodes in set *k*_*K*−1_, and the remaining nodes in set *k*_*i*_, for 1<*i*<*K*, receive links from nodes in *k*_*i*−1_ and send links to nodes in *k*_*i*+1_. Such input-output *K*-partite graph can be represented in the following matrix form

1 $$ A=\left(\begin{array}{ccccc} 0 & \qquad A_{1, 2} & \qquad0 & \qquad\dots & \qquad0 \\ 0 & \qquad0 & \qquad A_{2, 3} & \qquad\dots & \qquad0 \\ \vdots & \qquad\ddots & & \qquad\ddots & \qquad0 \\ 0 & \qquad\dots & & & \qquad A_{K-1, K}\\ 0 & \qquad\dots & & & \qquad 0 \end{array}\right),  $$

where *A*_*i,j*_ is the sub-matrix of the adjacency matrix *A* representing the links from nodes in set *k*_*i*_ to nodes in set *k*_*j*_, for 1≤*i*<*j*≤*K*. Thus, adjacency matrix *A* is a block matrix, with blocks *A*_*i,j*_. As an illustrative example, Fig. 1 shows an input-output tripartite graph in which the three sets correspond to distinct production stages and contain four, three, and four countries, respectively.

The adjacency matrix *W* corresponding to Fig. 1 is

2 $$ W=\left(\begin{array}{ccc} 0 & \begin{array}{ccc} \quad8 & \quad0 & \quad0 \\ \quad6 & \quad4 & \quad0 \\ \quad0 & \quad0 & \quad9 \\ \quad0 & \quad0 & \quad6 \\ \end{array} & \quad0 \\ 0 & \quad0 & \begin{array}{cccc} \quad4 & \quad0 & \quad0 & \quad0 \\ \quad10 & \quad6 & \quad0 & \quad0 \\ \quad0 & \quad0 & \quad7 & \quad11 \\ \end{array}\\ 0 & \quad0 & \quad0 \\ \end{array}\right).  $$

Unlike affiliation networks, in which each link relates an actor to a subset of events and each event to a subset of actors, the international GVN is dyadic since links relate countries with one another, albeit in different roles (Faust 1997). Correspondingly, the duality property of affiliation networks does not apply to the GVN as countries are not linked to one another through shared memberships of collectives, and collectives are not linked through shared countries. Rather, countries sharing connections to or from the same country may be seen as competing for the same customer or for the same supplier, respectively.

As in affiliation networks, however, in the GVN there should be centrality scores for each country belonging to each of the *K* sets. Furthermore, there should be some analytical relationships between these scores. That is, it is reasonable to assume that the centrality of a country *n*_*i*_ in set *k*_*i*_ is proportional to: (i) the centralities of the countries that are members of the set *k*_*i*−1_ from which *n*_*i*_ receives a connection, and (ii) the centralities of the countries in set *k*_*i*+1_ to which *n*_*i*_ directs a connection. Thus, as in the case of affiliation networks, focusing only on single sets of nodes (e.g., on a single stage of the production) would imply neglecting the fundamental relationships among sets of nodes across the whole network. Rather, the centrality of a country at a given production stage within a given industry should quantify the *salience of that country for the entire GVN*, and thus explicitly depend on the centrality of the other countries located at other production stages and with which the focal country is connected.

## The data

Our study draws upon the bilateral trade data set extracted from the BACI-CEPII database. The data set refers to the years 2007 and 2014, and includes 221 countries. Trade flows are recorded as 6-digit codes according to the Harmonized System (HS) classification (Revision 2002). We restricted our analysis to the trade flows in three industrial sectors: Electronics (HS02 Codes 85XXXX), Motor Vehicles (HS02 Codes 87XXXX), and Textiles and Apparel (HS02 Codes from 50XXXX to 63XXXX).

We then applied the Broad Economic Categories classification to assign each 6-digit code to one of the following three broad economic categories: “intermediate goods”, “consumer goods”, and “capital goods” (see Tables 1 and 2). We then aggregated consumer goods and capital goods into a broader “finished products” category. Finally, we used the two categories of intermediate goods and finished products to extract from the international trade network the international GVNs in which countries play distinct roles as exporters or importers at various stages of the production process (see details in the following section).

**Table 1:** USD values (current prices) and share of intermediate, consumer, and capital goods in the Electronics, Motor Vehicles, and Textiles and Apparel industries in 2007

2007		
Industry	World trade value	Share
Electronics	1,781,260,000	
Intermediate goods	1,050,100,000	59%
Consumer goods	235,910,000	13%
Capital goods	495,250,000	28%
Motor vehicles	1,184,322,000	
Intermediate goods	311,900,000	26%
Consumer goods	660,812,000	56%
Capital goods	211,610,000	18%
Textiles and Apparel	601,090,000	
Intermediate goods	217,920,000	36%
Consumer goods	383,170,000	64%

**Table 2:** USD values (current prices), share, and rate of variation of intermediate, consumer, and capital goods in the Electronics, Motor Vehicles, and Textiles and Apparel industries in 2014

2014				
Industry	World trade value	Change in value (%)	Share	Change in share (%)
Electronics	2,241,940,000	26%		
Intermediate goods	1,285,900,000	22%	57%	-3%
Consumer goods	232,250,000	-1%	10%	-22%
Capital goods	723,790,000	46%	32%	16%
Motor vehicles	1,364,500,000	15%		
Intermediate goods	402,230,000	29%	29%	12%
Consumer goods	733,480,000	11%	54%	-4%
Capital goods	228,760,000	8%	17%	-6%
Textiles and Apparel	753,750,000	25%		
Intermediate goods	262,210,000	20%	35%	-4%
Consumer goods	491,540,000	28%	65%	-2%

As indicated by Tables 1 and 2, between 2007 and 2014 the traded value of Motor Vehicles increased by 15%, which is less than the increase in the traded value in Electronics (25%) and in Textiles and Apparel (26%). Over the same period, the value of merchandise trade (including commodities) at world level increased by approximately 35%. Our period of observation includes the years in which trade was deeply affected by the international financial crisis, displaying large economic fluctuations, especially in sectors with a strong presence of GVCs, such as the ones considered here. In spite of this, no substantial change in trade composition can be observed between the two years analysed. Nevertheless, it is worth noting that in the Textiles and Apparel industry the finished products represent two thirds of the total trade in the same industry, with only a small difference between 2007 and 2014. The same can be observed in the Motor Vehicles sector. In particular, in Motor Vehicles, finished products toward final consumption represent more than 50% of the total trade in that industry in both years, with only a negligible decline in 2014. At the same time, trade of intermediate inputs increased between 2007 and 2014 while trade of finished products decreased, and trade of consumer goods remained lower than trade of capital goods in both years. By contrast, in Electronics trade remained concentrated more on intermediate inputs than finished products, and trade of capital goods was higher than trade of consumer goods in both 2007 and 2014, though between the two years the former increased while the latter decreased.

## Constructing the international global value networks

A necessary step toward the assessment of the centrality of countries in GVCs is represented by the extraction of the GVNs – i.e., the networks in which connections between countries are qualified as a function of the type of product traded – from the underlying international trade networks – i.e., the networks in which countries are connected to one another irrespective of the stage of production they occupy.

To this end, we define the tripartite valued graph whose nodes are partitioned into three different independent sets, *U*, *M*, and *D*, such that no two endpoints of a link belong to the same set, nor one to set *U* and the other to set *D*. The first set *U* refers to the population of exporters of intermediate inputs. The second set *M* refers to the population of countries that are importers of intermediate products or exporters of finished products or both importers of intermediate products and exporters of finished products. Finally, the third set *D* includes the importers of finished products.

So conceived of, only directed links connecting nodes in set *U* to nodes in set *M* and directed links connecting nodes in set *M* to nodes in set *D* are allowed. In particular, a directed link is established from a node in set *U* to a node in set *M* if the former node supplies intermediate inputs to the latter. Similarly, a directed link is established from a node in set *M* set to a node in set *D* if the former node supplies a finished product to the latter. Moreover, the weight of each link is given by the value in US dollars of the goods traded by the two connected countries.

In each set, each country can play an “active” or a “non-active” role. A country is active in a given set when it is involved in at least a trade flow of the nature defined by the set. For example, if a node is connected to the other nodes by only import trade flows of finished products, then it will be classified as “active” in set *D* and ”non-active” in the remaining two sets. The total number of countries *N* thus corresponds to the maximum number of “active” nodes each set can include. Moreover, in a given industry and a given year, each node in set *U* can have at most *N*−1 outgoing links pointing to nodes in set *M*. Each node in set *M* can have at most *N*−1 incoming links emanating from nodes in set *U* and *N*−1 outgoing links pointing to nodes in set *D*. Finally, each node in set *D* can receive at most *N*−1 incoming links from nodes in set *M*. That is, while a given country *n*_*i*_ can be member of all three sets, and therefore exert different roles at the same time in the GVN, it cannot point a link to itself in a different set.

Formally, we indicate with $w_{um}^{I}$ the outgoing weighted link representing the trade of intermediate inputs from node *u* belonging to set *U* to node *m* belonging to set *M*. Moreover, we indicate with $w_{md}^{F}$ the outgoing weighted link representing the trade of finished products from node *m* belonging to set *M* toward node *d* belonging to set *D*.

The tripartite network can be formalised in terms of the weighted matrix *W*_3−*p*_ in which the entries are defined as follows

3 $$ {}\left\{ \begin{array}{ll} w_{um}^{I}>0&\qquad\mathrm{if\:country\:}\mathit{u}\:\mathrm{exports\:intermediate\:products\:to\:country}\:\mathit{m;}\\ w_{md}^{F}>0&\qquad\mathrm{if\:country}\:\mathit{m}\:\mathrm{exports\:finished\:products\:to\:country}\:\mathit{d;}\\ w_{um}^{I}=w_{md}^{F}=0&\qquad\mathrm{otherwise.} \end{array}\right.  $$

Notice that in the international GVNs each country can play multiple roles simultaneously. For instance, within the same industry a country may import both intermediate and finished products, and at the same time also export both intermediate and finished products. So conceived of, the international GVNs thus capture the nuances of the GVCs, such as the exchange of the same intermediate or final products among different countries. In the case of intermediate products, when multiple transactions of intermediate products take place before the final products are exported, each country involved in such transactions would play the dual roles as exporter and importer of intermediate products, and thus would belong to both set *U* and set *M*. Similarly, in the case of multiple transactions of the same final products among countries, each country involved would play the roles as exporter and importer of such products, and would thus belong to both set *M* and set *D*.

The international GVN in a given industry can also be formalised as a two-layer multiplex network, in which the nodes are the countries, and each layer corresponds to one of two types of product traded between countries: intermediate and finished (De Domenico et al. 2015; Rahmede et al. 2017). Countries that belong to all three sets would also be part of both the intermediate-product layer and the finished-product layer. Countries that only belong to set *U* or only to set *D* would be nodes, respectively, in the intermediate-product layer or the finished-product layer. Countries belonging to set *M* that both import intermediate products and export finished ones would be nodes in both layers. Finally, countries in set *M* that only export finished products or only import intermediate products would be nodes only in the finished-product layer or in the intermediate-product layer, respectively.

## Formalising the centrality of countries in the international global value networks

The roles that countries occupy within the international GVNs can be unmasked through the application of suitable centrality measures to the international trade network of intermediate and finished products. To this end, here we propose the following three measures of centrality for capturing the degree to which a given country plays a prevailing role in the upstream, midstream, and downstream stages of the production in a given industry’s GVN:

- A country’s *upstreamness* centrality in an industry captures the tendency of the country to preferentially export intermediate goods to other countries that, in turn, have a tendency to preferentially export finished products and import intermediate inputs.
- A country’s *downstreamness* centrality in an industry captures the tendency of the country to preferentially import final products from countries that, in turn, tend to preferentially export finished products and import intermediate inputs.
- A country’s *midstreamness* centrality in an industry is a function of both the upstreamness of the countries from which the focal country imports the intermediate products and the downstreamness of the countries to which the focal country exports the finished products. More specifically, a country’s midstreamness centrality in an industry captures the tendency of the country to import intermediate goods preferentially from countries with high upstreamness centrality and to export final products preferentially to countries with high downstreamness centrality.

Given an industry, these three measures will be applied to the directed and weighted international global value tripartite network defined above, in which nodes are suitably labelled according to the roles they play as exporters or importers of intermediate or finished products.

We now proceed to formalise the three recursive measures of centrality. We begin by formalising the first-order midstreamness centrality, and we then formalise the first-order upstreamness and first-order downstreamness centralities as a function of first-order midstreamness centrality. In a similar way, we show how a *h*-order midstreamness centrality can be defined in terms of the *h*−1-order upstreamness and *h*−1-order downstreamness centralities. The three centralities then converge to higher-order values that are the solutions of a linear system of equations.

First, we define the *first-order midstreamness* centrality of a country as the sum of the intermediate inputs imported and the finished products exported by the country. This measure thus broadly captures the country’s centrality as an *intermediary actor* in the production of products in a certain industry. Formally, a country *m* in set *M* can be characterised by the sum of the weights of all its incoming and outgoing links, i.e., the total *strength* *s*_*m*_, as follows

4 $$ \mu_{m}^{1}=s_{m}=\sum_{u\in U}w_{um}^{I}+\sum_{d\in D}w_{md}^{F}.  $$

To normalise the first-order midstreamness centrality of each country, we divide Eq. 4 by the maximum midstreamness value across all countries

5 $$ \frac{\mu_{m}^{1}} {\mu^{*}} \times 100,  $$

where $\mu ^{*}=\underset {m}{max}\,(\mu _{m}^{1})$.

Next, the *first-order upstreamness* centrality of a country *u* included in set *U* is defined as the *weighed sum* of the values of its outgoing links representing the exports of intermediate inputs toward countries in set *M*, where the weight of the value of each link in the sum is the first-order midstreamness centrality $\mu _{m}^{1}$ of the country *m* in set *M* to which the link is directed. Formally, we define the first-order upstreamness centrality of a country *u* in set *U* as follows

6 $$ \upsilon_{u}^{1}=\sum_{m\in M}\mu_{m}^{1}w_{um}^{I}.   $$

To normalise the first-order upstreamness centrality of each country, we then divide Eq. 6 by the maximum upstreamness value across all countries

7 $$ \frac{\upsilon_{u}^{1}} {\upsilon^{*}} \times 100,   $$

where $\upsilon ^{*}=\underset {u}{max}\,(\upsilon _{u}^{1})$.

The *first-order downstreamness* centrality of a country *d* included in set *D* is instead defined as the *weighed sum* of the values of the country’s incoming links representing the imports of finished products from countries in set *M*, where the weight of the value of each link in the sum is the first-order midstreamness centrality $\mu _{m}^{1}$ of the country *m* in set *M* from which the link originates. Formally, we define the first-order downstreamness centrality of a country *d* in set *D* as follows

8 $$ \delta_{d}^{1}=\sum_{m\in M}\mu_{m}^{1}w_{md}^{F}.   $$

To normalise the first-order downstreamness centrality of each country, we then divide Eq. 8 by the maximum downstreamness value across all countries

9 $$ \frac{\delta_{d}^{1}} {\delta^{*}} \times 100,   $$

where $\delta ^{*}=\underset {d}{max}\,(\delta _{d}^{1})$.

Next, we define the *second-order midstreamness* centrality of a country *m* in set *M* as the *weighted sum* of the values of the links that *m* receives from countries in set *U* and directs to countries in set *D*, where the weights in the sum of the values of the links from countries in set *U* and to countries in set *D* are, respectively, the first-order upstreamness centralities of the countries in *U* and the first-order downstreamness centralities of the countries in *D*. Formally, we define the second-order midstreamness centrality of a country *m* in set *M* as

10 $$ \mu_{m}^{2}=\sum_{u\in U}\upsilon_{u}^{1}w_{um}^{I}+\sum_{d\in D}\delta_{d}^{1}w_{md}^{F}.   $$

To normalise the second-order midstreamness centrality of each country, we then divide Eq. 10 by the maximum value of midstreamness across all countries

11 $$ \frac{\mu_{m}^{2}} {\mu^{*}} \times 100,   $$

where $\mu ^{*}=\underset {m}{max}\,(\mu _{m}^{2})$.

Finally, the *higher-order* upstreamness, downstreamness, and midstreamness centrality measures are the solutions of the following linear system

12 $$ \left\{ \begin{array}{l} \upsilon_{u}^{h}=\sum_{m\in M}\mu_{m}^{h}w_{um}^{I}\\ \delta_{d}^{h}=\sum_{m\in M}\mu_{m}^{h}w_{md}^{F}\\ \mu_{m}^{h}=\sum_{u\in U}\upsilon_{u}^{h-1}w_{um}^{I}+\sum_{d\in D}\delta_{d}^{h-1}w_{md}^{F} \end{array}\right.  $$

All countries that are involved in at least one transaction (exports or imports) of (intermediate or finished) products will be assigned at least one value of the centralities defined above. In the previous section we mentioned that the international GVNs capture the nuances of international trade, such as chains of multiple transactions of intermediate or finished products. In particular, two cases can be identified. First, a country may well control the whole production of a given product, and contribute to the exports without importing any input. Second, a country may play a prominent role in many stages of the transformation process without necessarily exporting the final product. In the former case, the country that exports finished products but does not import intermediate ones would still occupy the midstream stage of the GVN and thus be classified as part of set *M*. However, since there would be no incoming links pointing to the country, its midstreamness centrality would not be defined in terms of the upstreamness of other countries. Similarly, in the latter case, the country that exports and imports only intermediate products would occupy both an upstream and a midstream stage in the GVN, and would thus be classified as a member of both set *U* and set *M*. However, since there are no outgoing flows of finished products from that country toward other countries, its midstreamness centrality would not be defined in terms of the downsteamness centralities of the other countries.

## Results

**Fig. 2:**
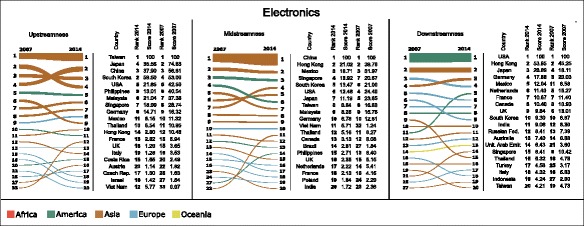
Bump charts showing the variation in rankings and values of upstreamness (*left-hand column*), midstreamness (*centre column*), and downstreamness (*right-hand column*) centralities of countries in the Electronics industry between 2007 and 2014. Each line in the bump charts shows the variation of ranking for each country, and the width of each line at the two endpoints is proportional to the centrality score in the corresponding year. Only the 20 top-ranked countries in 2014 have been included. The scores underlying the two rankings in each bump chart have been normalised so as to produce equal sums of widths of endpoints at each year. Beside each bump chart is a table illustrating the rankings and centrality scores normalised according to Eqs. 5, 7, and 9. The colour of each line in the bump charts refers to the geographic continent of the corresponding country. Bump charts were obtained using RAWGraphs

**Fig. 3:**
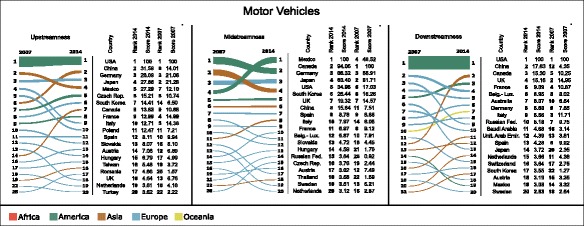
Bump charts showing the variation in rankings and values of upstreamness (*left-hand column*), midstreamness (*centre column*), and downstreamness (*right-hand column*) centralities of countries in the Motor Vehicles industry between 2007 and 2014. Each line in the bump charts shows the variation of ranking for each country, and the width of each line at the two endpoints is proportional to the centrality score in the corresponding year. Only the 20 top-ranked countries in 2014 have been included. The scores underlying the two rankings in each bump chart have been normalised so as to produce equal sums of widths of endpoints at each year. Beside each bump chart is a table illustrating the rankings and centrality scores normalised according to Eqs. 5, 7, and 9. The colour of each line in the bump charts refers to the geographic continent of the corresponding country. Bump charts were obtained using RAWGraphs

**Fig. 4:**
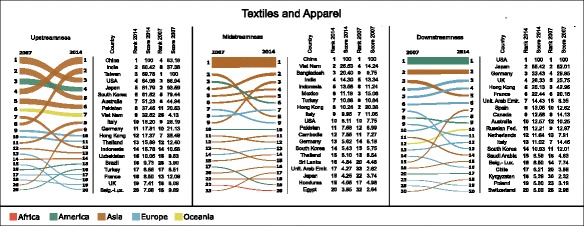
Bump charts showing the variation in rankings and values of upstreamness (*left-hand column*), midstreamness (*centre column*), and downstreamness (*right-hand column*) centralities of countries in the Textiles and Apparel industry between 2007 and 2014. Each line in the bump charts shows the variation of ranking for each country, and the width of each line at the two endpoints is proportional to the centrality score in the corresponding year. Only the 20 top-ranked countries in 2014 have been included. The scores underlying the two rankings in each bump chart have been normalised so as to produce equal sums of widths of endpoints at each year. Beside each bump chart is a table illustrating the rankings and centrality scores normalised according to Eqs. 5, 7, and 9. The colour of each line in the bump charts refers to the geographic continent of the corresponding country. Bump charts were obtained using RAWGraphs

Figures 2, 3, and 4 show the variation in the rankings and values (normalised according to Eqs. 5, 7, and 9) of upstream (left-hand column), midstream (centre column), and downstream (right-hand column) centralities of countries, respectively in Electronics, Motor Vehicles, and Textile and Apparel, between 2007 and 2014. For the sake of simplicity, the figures restrict their focus only to the 20 top-ranked countries in 2014, and show which positions these countries held in 2007.

**Fig. 5:**
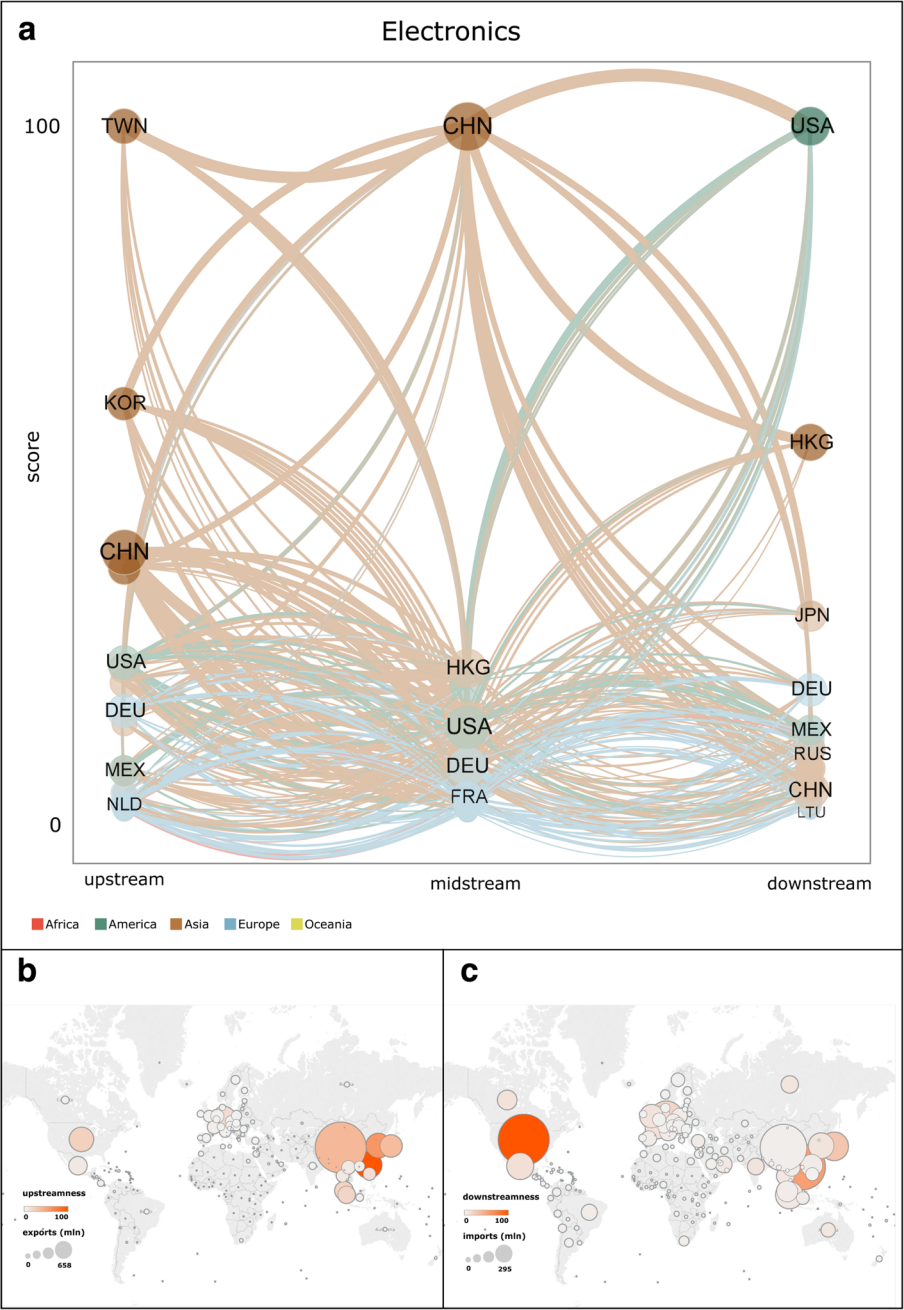
Rankings and values of upstreamness centrality, midstreamness centrality, downstreamness centrality, exports, and imports of countries in the Electronics industry in 2014. In panel (**a**), the size of each node (country) in the network is proportional to the sum of the country’s exports of intermediate inputs (upstream), the sum of the country’s imports of intermediate inputs and exports of final products (midstream), and the sum of the country’s imports of final products (downstream). For each centrality measure, countries are ranked according to the corresponding score (highest at the top). Each directed link connects a country in set *U* to a country in set *M*, or a country in set *M* to a country in set *U*, according to the definition of the tripartite network given in the text. The width of each link is proportional to the value of products exchanged by the two connected countries. The colour of each link refers to the continent of the country from which the link originates. Panels (**b**) and (**c**) show the geographic map in which each country is represented as a circle whose diameter is proportional to the country’s total exports (panel (**b**)) and total imports (panel (**c**)), and whose colour varies according to the corresponding value of upstreamness (panel (**b**)) and downstreamness (panel (**c**)) centralities. The network in panel (**a**) was visualised through VOS Viewer. Maps in panels (**b**) and (**c**) were visualised using Tableau Software

**Fig. 6:**
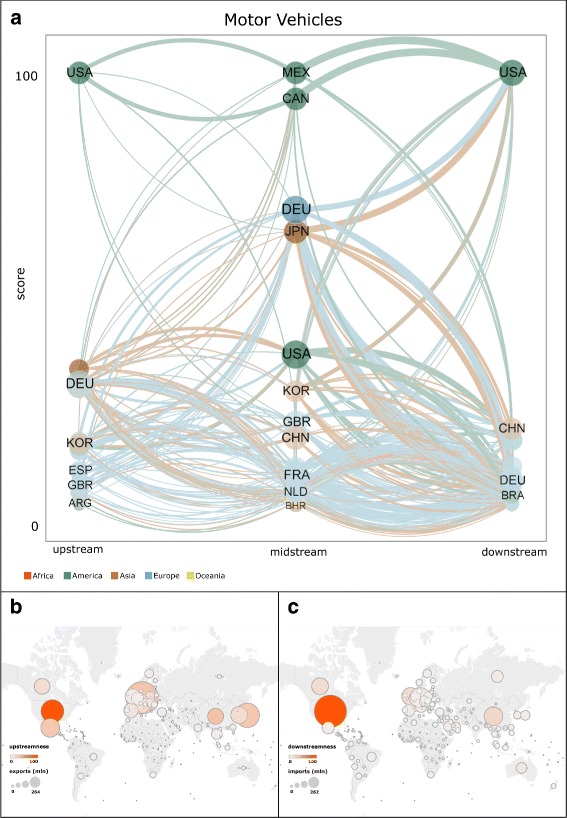
Rankings and values of upstreamness centrality, midstreamness centrality, downstreamness centrality, exports, and imports of countries in the Motor Vehicles industry in 2014. In panel (**a**), the size of each node (country) in the network is proportional to the sum of the country’s exports of intermediate inputs (upstream), the sum of the country’s imports of intermediate inputs and exports of final products (midstream), and the sum of the country’s imports of final products (downstream). For each centrality measure, countries are ranked according to the corresponding score (highest at the top). Each directed link connects a country in set *U* to a country in set *M*, or a country in set *M* to a country in set *U*, according to the definition of the tripartite network given in the text. The width of each link is proportional to the value of products exchanged by the two connected countries. The colour of each link refers to the continent of the country from which the link originates. Panels (**b**) and (**c**) show the geographic map in which each country is represented as a circle whose diameter is proportional to the country’s total exports (panel (**b**)) and total imports (panel (**c**)), and whose colour varies according to the corresponding value of upstreamness (panel (**b**)) and downstreamness (panel (**c**)) centralities. The network in panel (**a**) was visualised through VOS Viewer. Maps in panels (**b**) and (**c**) were visualised using Tableau Software

**Fig. 7:**
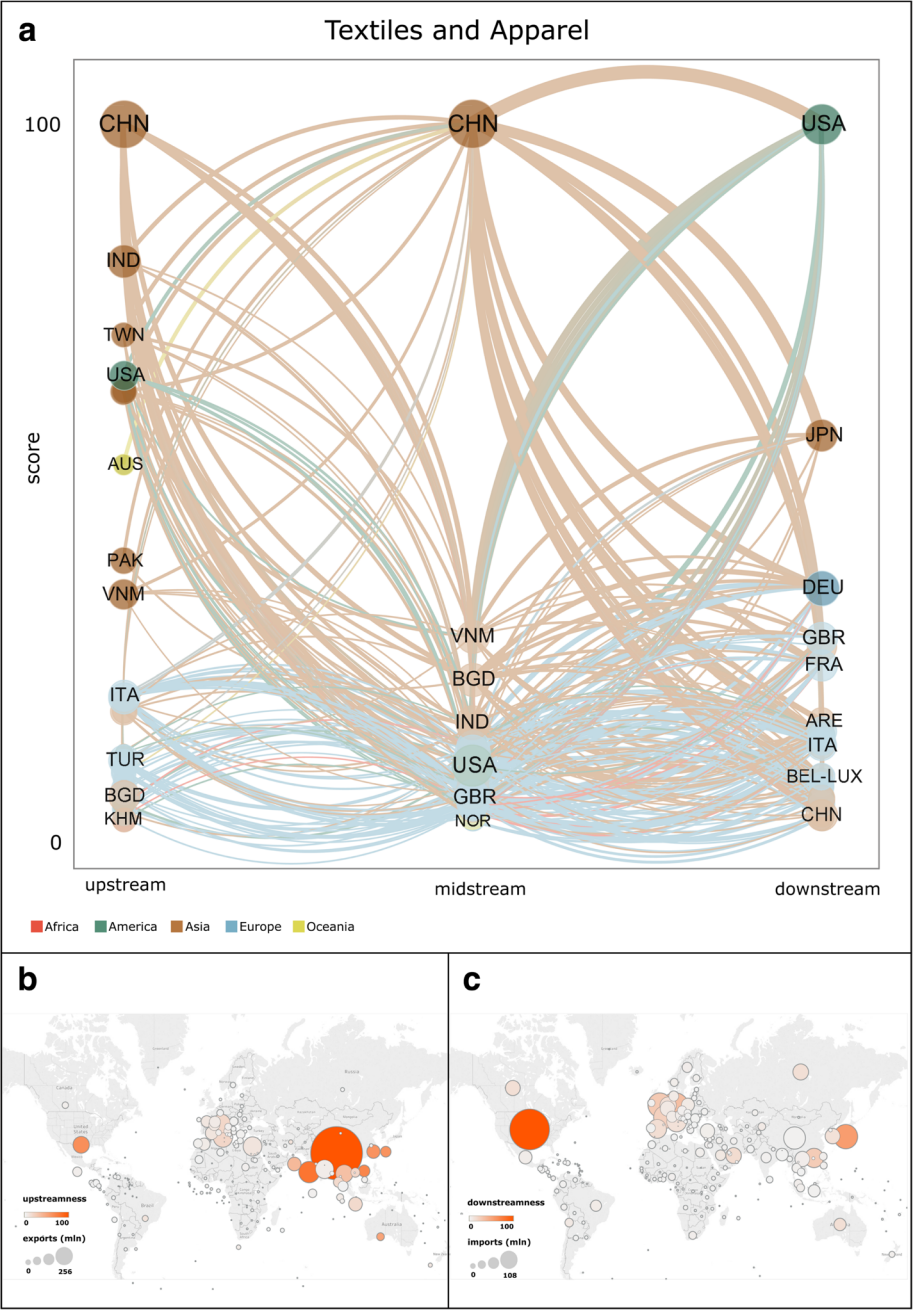
Rankings and values of upstreamness centrality, midstreamness centrality, downstreamness centrality, exports, and imports of countries in the Textiles and Apparel industry in 2014. In panel (**a**), the size of each node (country) in the network is proportional to the sum of the country’s exports of intermediate inputs (upstream), the sum of the country’s imports of intermediate inputs and exports of final products (midstream), and the sum of the country’s imports of final products (downstream). For each centrality measure, countries are ranked according to the corresponding score (highest at the top). Each directed link connects a country in set *U* to a country in set *M*, or a country in set *M* to a country in set *U*, according to the definition of the tripartite network given in the text. The width of each link is proportional to the value of products exchanged by the two connected countries. The colour of each link refers to the continent of the country from which the link originates. Panels (**b**) and (**c**) show the geographic map in which each country is represented as a circle whose diameter is proportional to the country’s total exports (panel (**b**)) and total imports (panel (**c**)), and whose colour varies according to the corresponding value of upstreamness (panel (**b**)) and downstreamness (panel (**c**)) centralities. The network in panel (**a**) was visualised through VOS Viewer. Maps in panels (**b**) and (**c**) were visualised using Tableau Software

Figures 5, 6, and 7 show the rankings and values of upstreamness centrality, midstreamness centrality, downstreamness centrality, exports, and imports of countries in 2014, respectively in the Electronics, Motor Vehicles, and Textiles and Apparel industries. In panel (a) in all these figures, the size of each node (country) in the tripartite network is proportional to: (i) the sum of the country’s exports of intermediate inputs (upstream); (ii) the sum of the country’s imports of intermediate inputs and exports of final products (midstream); and (iii) the sum of the country’s imports of final products (downstream). At each production step of the GVN in a given industry, countries are then ranked according to the corresponding centrality score (highest at the top). Panels (b) and (c) of the same figures show the geographic map in which each country is represented as a circle whose diameter is proportional to the country’s total exports (panel (b)) and total imports (panel (c)), and whose colour varies according to the corresponding value of upstreamness (panel (b)) and downstreamness (panel (c)) centralities.

Additional file 1: Tables S1, S2, and S3 report the top 50 countries ranked according to the exports in 2014 in the Electronics, Motor Vehicles, and Textiles and Apparel industries, respectively. In all these tables, alternative rankings based on, and the corresponding values of, imports in 2014, and upstreamness centrality, midstreamness centrality, and downstreamness centrality in 2007 and 2014 are also reported.

The positions that countries occupy in the international GVNs are not only determined by the sheer size of the countries in terms of potential output (given by the amount of labour force, physical capital and technology available) or domestic demand (given by population and income). Certainly, the economic size of countries is relevant for it allows them to expand their production and trade, but it is not the only factor affecting the countries’ centrality in the international production networks. This can be observed in Figs. 5, 6, and 7, where the size of countries measured in terms of exports and imports is compared to their centrality scores. Exports, if normalised, are equivalent to a country’s share of the world’s total exports in a given sector, and it is highly correlated with the economic size in terms of gross domestic product of countries (De Benedictis et al. 2014). Also the correlation between exports (imports) and our centrality measures is positive, but far from perfect. For example, in the case of Electronics, Fig. 5 suggests that China is the second-ranked country in terms of imports, but it is not equally central as a final market for these goods since it holds a much lower-ranked position in downstream centrality. Somewhat similar profiles are the ones of Taiwan and Malaysia, countries that import many electronic intermediate products but are not equally relevant as a final market (see also Fig. 2). On the contrary, countries such as Mexico and Brazil are more central in the intermediate positions of the international production network of electronic goods than their trade volumes would lead us to expect, while many advanced European countries are much less central. As indicated by Fig. 6, in the Motor Vehicles industry some small countries such as the Czech Republic and Austria, tightly connected to the German production chain, maintain very central positions especially at the upstream and midstream stages where, respectively, they produce and export components, and import components and export final goods (see also Fig. 3). The industry where the correlation between the more traditional measures of trade and our measures of centrality is generally lower is the Textile and Apparel, a traditional industry where also small and less developed countries can play relevant roles at various stages of the production (Fig. 7).

The observed deviations between a standard measure of relevance in trade, such as exports or imports, and our indicators imply that our centrality measures capture the economic relevance of a country in a given sector beyond its market share in that sector. In particular, the overall results that we obtained provide empirical support in favour of many of the hypotheses offered by recently proposed economic models on GVCs: different countries hold the most central positions at different stages of the production process. Emerging and developing countries tend to be more central in the midstream production phases. In the Textiles and Apparel sector, Bangladesh is the third most central country, as it is a very relevant location for the production and assembly of many apparel intermediate goods (see Fig. 4). However, Bangladesh does not seem to control the organisation of production of these goods, as its relative centrality in the upstream position is much lower. In the same sector, the highest downstream centrality is held by more advanced countries (e.g., USA, Japan, Germany), whose large domestic markets and important distribution chains allow them to maintain these positions. As indicated by Figs. 2, 3, and 4, this pattern characterises all sectors: high-income countries with large markets tend to secure a higher value of downstreamness centrality than low-income countries.

The GVN in Electronics, especially in the upstream and midstream positions, is dominated by East Asian countries that appear indeed as the world’s “factory” in this sector (see Fig. 3). But in another technologically advanced sector such as Motor Vehicles, midstream central positions too are held by relatively advanced countries (see Fig. 4). The Motor Vehicles industry is still organised around some European and North American countries, but it is highly internationalised as a number of small countries play a relevant role at various stages of the corresponding GVN.

We also note that the structure of the international production networks is organised differently across sectors, in qualitative agreement with what has been suggested by other studies of sectoral trade networks (Cingolani et al. 2015). The production in the Textiles and Apparel sector is relatively less centralised, with groups of countries holding similar positions (see Fig. 4 and panels (b) and (c) in Fig. 7). In the Electronics and Motor Vehicles industries, the number of countries with very high centrality is smaller and more geographically concentrated within specific areas (see Fig. 2 and panels (b) and (c) in Figs. 5 and 6, respectively). This is what would be expected in sectors where technological knowledge is important for production and this knowledge is concentrated only within a minority of countries.

Our findings also suggest that over time many emerging countries strengthened their participation in GVNs. China is by far the most central country in the intermediate parts of the Textiles and Electronics industries, and its position has increased at the upstream production stage in the Motor Vehicles and Textiles industries (see Figs. 2, 3, and 4). But besides the particular case of China, our results indicate that the upstreamness centrality of other smaller countries in East Asia, such as Vietnam, and in parts of Central Europe and South America, has also increased over time.

## Conclusions

The international production of goods and services is increasingly organised along GVCs in which the various production tasks are performed at many different locations all over the world. Typically, production is not co-extensive with final consumption. Indeed intermediate goods produced in one country can be further transformed by many other countries, and follow complex chains of production stages (at which the same country may, in principle, be involved multiple times) before they are exported and finally consumed in the destination markets. Thus, the market power that a country exerts in global trade cannot be properly assessed simply by relying on traditional trade statistics constructed from gross trade values (Lejour et al. 2014).

Centrality in global trade is important insofar as it enables a country to have a large number of economic transactions with alternative potential suppliers and wide access to different important markets. However, within GVNs the role of centrality changes according to which phase of the production process each country occupies. Recent economic literature on trade in value added has described upstream and downstream positions as more advantageous sources of bargaining power that strengthen countries’ ability to set prices than other intermediate positions in the production process (Johnson and Noguera 2012). High centrality in upstream phases enables a country to control key inputs to the production process, while high centrality in the downstream phases enables a country to remain close to the final demand and thus to control final prices in the destination markets (Antràs et al. 2012). To fully assess a country’s position in the international production of goods and services, it is therefore crucial to evaluate a country’s centrality at the various stages into which the GVCs can be articulated (Li et al. 2014).

Our study was an attempt to pave the way in this direction. To this end, we proposed a novel three-faceted measure of centrality that captures a country’s distinct roles at the upstream, midstream, and downstream stages of the international production process. Underlying the formalisation of our centrality measure was the construction of the international GVNs – in which connections between countries are qualified in terms of the type of product traded – from the aggregate trade network – in which no distinction is made between different stages of production. The GVNs were then formalised as tripartite weighted and directed graphs in which nodes (i.e., countries) are partitioned into three different independent sets, each representing a production stage.

We computed our three recursive measures of upstreamness, midstreamness, and downstreamness centrality using the bilateral trade data set extracted from the BACI-CEPII database, and to which the Broad Economic Categories classification was applied to distinguish between intermediate and finished products. We restricted our analysis to three industries (i.e., Electronics, Motor Vehicles, and Textiles and Apparel), and two years (i.e., 2007 and 2014). Our findings suggest that countries hold different positions at the various stages of the international production process, and these positions change over time. Moreover, these variations in centrality according to the roles countries occupy along the GVNs would remain undetected if more traditional measures of market power based on aggregate trade values were used. In the Electronics industry, for example, China and Taiwan are highly ranked in terms of aggregate gross imports, and yet they do not hold an equally highly ranked position at the downstream production stage (see Additional file 1: Table S1).

More generally, even though rankings of countries based on our centrality measures are somehow correlated with rankings based on more traditional measures of trade, these correlations tend to weaken when focus is restricted to the top-ranked countries. Indeed the top countries according to traditional measures do not necessarily occupy the same positions when ranked according to any of our centrality measures. Moreover, our analysis suggests that these differences between rankings are likely to amplify within more traditional industries in which smaller and less developed countries can secure a relatively central position, as is the case of Textiles and Apparel.

Our study contributes to recent debates on trade in value added generated in GVCs, and based on national input–output tables and international trade statistics (Lejour et al. 2014). Our proposed recursive indicators extend previous studies in that they allow us to clearly assess a country’s centrality at a distinct stage in the global production networks as a function of the centrality of other countries at other stages. Our findings may assist policy-makers in their efforts to improve countries’ international positions and competitive advantage. Centrality in an international production system is not only a function of how upstream or downstream a country is positioned along a linear sequence of production steps. Indeed, by simply moving along a GVC, a country does not necessarily improve its centrality and market power. By contrast, our analysis shows that choosing the appropriate suppliers and destination markets can have more important implications for the centrality of a country than the simple pursuit of large trade volumes at a given stage of the production process. From this perspective, our results can also inform policy-makers as they select which countries, given their initial positions in the GVNs, are most suitable for signing successful trade agreements.

In addition to the GVNs, our centrality measures can also be applied to other empirical settings in which the data can be represented as input-output *K*–partite graphs with *K* distinct sets of nodes. This is the case, for example, of inter-firm networks in which the firms are connected along serial supply chains within or across countries (Coe et al. 2008), or innovation networks in which pools of knowledge can combine and be transformed along generative processes leading to the production of new knowledge (Ernst and Kim 2002; Renoust et al. 2017). Similarly, the process of financial intermediation between savers and borrowers lends itself to the analysis of the centrality of the economic actors located at the various stages of the financial processes (Battiston et al. 2003). Alternatively, our measures can capture the role that banks play in the interbank lending market as a function of the position they occupy at the various stages of the lending process (Bardoscia et al. 2017; Battiston et al. 2012).

Our analysis is not without its limitations, and can be further extended along a number of avenues. First, the formalisation of centrality was based on the definition of the GVN as a tripartite graph, thus representing the production process as organised into three distinct stages. Longer sequences of production stages can be accounted for, and a more refined centrality measure can be formalised so as to reflect the positions of countries, located halfway along the transformation process, that both import and export intermediate inputs. Second, while our analysis was restricted to two years, a longer observation period can certainly provide useful insights into the patterns of evolution of centrality over time. Finally, future work can be extended beyond the three industries here investigated, and can thus contribute toward a comparative assessment of GVNs involving various proportions of low- and high-skilled labour and of capital.

## Additional file

Additional file 1 Appendix. (ZIP 258 kb)
