# Supplementary material for: Countries’ positions in the international global value networks: Centrality and economic performance
Source: Appl Netw Sci. 2017 Jul 12;2(1):21. doi: 10.1007/s41109-017-0041-4 (PMC6214273; doi:10.1007/s41109-017-0041-4)
Supplement: Supplementary file 1 — Appendix. (ZIP 258 kb) [file 41109_2017_41_MOESM1_ESM.zip › TableS2.pdf]

Motor Vehicles																	
Countries	Continent	**Rank Exports 2014**	Exports ($M) 2014	Rank Imports 2014	Imports ($M) 2014	Rank Upstreamness 2014	Upstreamness 2014	Rank Upstreamness 2007	Upstreamness 2007	Rank Midstreamness 2014	Midstreamness 2014	Rank Midstreamness 2007	Midstreamness 2007	Rank Downstreamness 2014	Downstreamness 2014	Rank Downstreamness 2007	Downstreamness 2007
Germany	Europe	**1**	263,941	2	103,893	3	28.09	3	21.08	3	68.32	3	55.91	8	5.68	9	7.55
Japan	Asia	**2**	149,280	14	21,717	4	27.66	2	21.28	4	63.40	2	81.71	14	3.72	20	2.35
USA	America	**3**	130,419	1	262,451	1	100.00	1	100.00	5	34.95	5	17.03	1	100.00	1	100.00
Mexico	America	**4**	88,069	8	35,561	5	27.29	7	12.10	1	100.00	4	48.52	19	3.03	14	3.32
South Korea	Asia	**5**	70,252	24	13,336	7	14.41	14	6.50	6	26.44	6	16.26	17	3.55	32	1.27
China	Asia	**6**	62,145	3	86,277	2	31.59	6	14.01	8	15.64	11	7.51	2	17.83	12	4.35
Canada	America	**7**	59,925	5	69,677	8	13.83	9	10.68	2	94.05	1	100.00	3	15.30	5	10.25
UK	Europe	**8**	57,603	4	77,140	18	4.64	13	6.76	7	19.32	7	14.57	4	15.16	2	14.95
France	Europe	**9**	51,392	6	59,035	9	12.99	4	14.99	11	6.97	8	9.12	5	9.09	4	10.87
Spain	Europe	**10**	50,491	11	32,150	12	8.11	10	9.94	9	8.78	9	8.86	13	4.28	6	9.92
Italy	Europe	**11**	38,656	9	34,627	10	12.71	5	14.38	10	7.97	14	6.05	9	5.56	3	11.71
Belg.- Lux.	Europe	**12**	37,083	7	43,592	21	3.26	15	5.22	12	6.87	10	7.91	6	6.95	8	8.02
Czech Republic	Europe	**13**	32,413	23	13,884	6	15.21	8	10.74	16	3.76	19	2.44	33	1.14	35	1.15
Thailand	Asia	**14**	26,660	30	8,247	22	3.10	21	2.50	18	3.58	22	1.59	54	0.48	50	0.64
Poland	Europe	**15**	21,690	18	18,017	11	12.47	11	7.21	22	2.96	18	2.53	23	2.00	19	2.41
Slovakia	Europe	**16**	21,329	26	9,476	13	8.07	16	5.10	13	4.72	15	4.45	61	0.45	56	0.51
Turkey	Europe	**17**	19,145	21	16,056	20	3.52	22	2.22	21	3.10	17	2.61	21	2.55	24	1.76
Sweden	Europe	**18**	18,097	20	16,400	23	2.93	20	3.26	19	3.51	13	6.21	20	2.83	18	2.64
Netherlands	Europe	**19**	17,674	13	25,548	19	3.81	18	4.18	20	3.12	16	2.87	15	3.66	11	4.38
Hungary	Europe	**20**	16,732	25	9,604	15	6.79	17	4.99	14	4.59	21	1.70	41	0.80	37	1.10
Austria	Europe	**21**	14,481	19	17,285	14	7.06	12	6.89	17	3.62	12	7.49	18	3.19	15	3.25
India	Asia	**22**	14,089	48	4,878	24	2.86	27	1.17	25	2.09	29	0.83	101	0.11	82	0.19
Brazil	America	**23**	10,295	16	19,666	26	1.63	23	1.92	24	2.82	20	1.79	22	2.31	48	0.68
romania	Europe	**24**	9,064	39	6,118	17	4.66	25	1.57	30	1.08	37	0.38	48	0.55	30	1.31
Taiwan	Asia	**25**	8,875	33	7,324	16	5.48	19	3.72	26	2.01	24	1.35	28	1.61	44	0.87
Argentina	America	**26**	8,394	29	8,651	35	0.46	35	0.57	33	0.91	32	0.44	49	0.54	63	0.41
South Africa	Africa	**27**	8,242	27	9,451	34	0.54	32	0.94	23	2.92	23	1.44	25	1.78	21	2.25
Portugal	Europe	**28**	6,581	32	7,610	29	1.38	26	1.45	28	1.29	26	1.29	36	0.99	33	1.20
Indonesia	Asia	**29**	5,706	38	6,537	27	1.49	29	1.04	31	1.04	35	0.39	57	0.46	57	0.51
Slovenia	Europe	**30**	4,264	56	3,481	30	1.25	30	1.03	45	0.41	31	0.56	60	0.46	51	0.59
Finland	Europe	**31**	3,435	44	5,322	41	0.20	41	0.21	35	0.61	27	1.05	38	0.91	23	1.79
Russian Federation	Europe	**32**	3,227	10	33,447	49	0.08	56	0.04	15	3.84	28	0.92	10	5.18	7	9.75
Morocco	Africa	**33**	2,824	57	3,339	56	0.04	57	0.04	44	0.41	63	0.07	62	0.40	60	0.44
Australia	Oceania	**34**	2,684	12	26,508	36	0.43	38	0.46	27	1.78	25	1.32	7	6.07	10	6.54
Switzerland	Europe	**35**	2,616	22	15,216	25	2.09	24	1.79	37	0.54	38	0.37	16	3.64	17	2.75
Belarus	Europe	**36**	2,522	77	1,374	54	0.05	62	0.02	52	0.21	42	0.27	102	0.10	80	0.23
Denmark	Europe	**37**	2,231	34	7,176	31	0.91	31	0.96	42	0.42	39	0.36	34	1.13	26	1.62
Singapore	Asia	**38**	2,034	47	5,027	38	0.30	40	0.27	32	0.97	34	0.40	43	0.76	40	1.05
Malaysia	Asia	**39**	1,894	36	6,733	32	0.73	37	0.48	34	0.78	40	0.29	39	0.91	46	0.74
Viet Nam	Asia	**40**	1,823	52	4,473	28	1.45	34	0.62	36	0.60	53	0.14	59	0.46	78	0.25
Hong Kong	Asia	**41**	1,537	46	5,050	42	0.15	49	0.07	41	0.46	49	0.19	32	1.16	43	0.87
United Arab Emirates	Asia	**42**	1,420	17	18,221	58	0.04	59	0.03	29	1.20	30	0.68	12	4.39	13	3.81
Philippines	Asia	**43**	1,133	40	5,674	33	0.69	28	1.17	50	0.26	51	0.15	46	0.68	74	0.28
Lithuania	Europe	**44**	1,078	62	2,302	44	0.13	43	0.12	66	0.12	54	0.12	66	0.32	53	0.57
Kuwait	Asia	**45**	961	49	4,875	73	0.00	68	0.01	53	0.19	55	0.11	30	1.39	36	1.11
Norway	Europe	**46**	869	28	9,053	37	0.41	33	0.69	49	0.28	43	0.24	24	1.98	22	2.06
Estonia	Europe	**47**	855	75	1,476	46	0.11	50	0.06	81	0.07	70	0.05	74	0.26	59	0.45
Bulgaria	Europe	**48**	687	67	2,047	45	0.13	54	0.05	67	0.11	68	0.06	70	0.30	55	0.53
Chile	America	**49**	650	31	7,640	55	0.04	44	0.12	43	0.41	46	0.21	29	1.59	34	1.18
Bahrain	Asia	**50**	637	69	1,829	61	0.03	78	0.00	70	0.10	94	0.02	50	0.53	61	0.43
