# Supplementary material for: Countries’ positions in the international global value networks: Centrality and economic performance
Source: Appl Netw Sci. 2017 Jul 12;2(1):21. doi: 10.1007/s41109-017-0041-4 (PMC6214273; doi:10.1007/s41109-017-0041-4)
Supplement: Supplementary file 1 — Appendix. (ZIP 258 kb) [file 41109_2017_41_MOESM1_ESM.zip › TableS3.pdf]

Textiles and Apparel																	
Countries	Continent	Rank Exports 2014	Exports ($M) 2014	Rank Imports 2014	Imports ($M) 2014	Rank Upstreamness 2014	Upstreamness 2014	Rank Upstreamness 2007	Upstreamness 2007	Rank Midstreamness 2014	Midstreamness 2014	Rank Midstreamness 2007	Midstreamness 2007	Rank Downstreamness 2014	Downstreamness 2014	Rank Downstreamness 2007	Downstreamness 2007
China	Asia	**1**	256,819	6	31,386	1	100.00	4	83.18	1	100.00	1	100.00	57	1.01	50	0.68
India	Asia	**2**	39,363	28	6,292	2	80.42	8	37.38	4	14.30	5	13.34	48	1.34	70	0.35
Italy	Europe	**3**	33,093	7	26,339	10	18.20	9	28.19	9	9.95	7	11.06	13	11.02	7	14.45
Turkey	Europe	**4**	31,314	17	13,224	17	8.86	17	8.51	7	10.88	8	10.84	30	2.66	36	1.78
Germany	Europe	**5**	30,753	2	54,453	11	17.81	10	21.13	13	5.62	14	6.18	3	33.43	4	29.95
Bangladesh	Asia	**6**	30,055	20	9,359	28	3.72	37	2.50	3	20.40	9	9.75	65	0.66	95	0.15
Viet Nam	Asia	**7**	26,770	15	14,167	9	32.62	26	4.13	2	26.53	4	14.24	25	3.42	59	0.47
USA	America	**8**	23,735	1	108,434	4	64.08	3	86.94	10	8.11	10	7.75	1	100.00	1	100.00
Indonesia	Asia	**9**	15,701	23	8,885	14	15.78	14	10.58	5	13.58	6	11.24	52	1.19	46	0.95
Pakistan	Asia	**10**	15,434	44	3,247	8	37.45	11	20.53	11	7.68	12	6.99	80	0.36	63	0.45
South Korea	Asia	**11**	15,134	14	14,424	6	61.62	5	79.44	14	5.43	15	5.75	14	10.93	11	12.01
France	Europe	**12**	13,921	5	32,884	18	8.50	13	12.09	22	3.61	18	4.67	6	22.44	6	20.16
Spain	Europe	**13**	11,889	9	22,643	27	4.36	20	6.05	29	2.75	27	2.78	8	13.08	10	12.62
Netherlands	Europe	**14**	11,286	10	18,235	25	4.80	21	5.70	26	3.24	31	2.69	12	11.64	13	7.91
Taiwan	Asia	**15**	11,067	39	3,524	3	69.78	1	100.00	41	1.86	25	2.86	33	2.56	40	1.65
Belg.- Lux.	Europe	**16**	10,884	13	14,810	20	7.08	15	9.69	28	2.83	21	3.85	16	6.50	14	7.74
UK	Europe	**17**	10,550	4	37,317	19	7.41	18	8.09	25	3.44	23	3.48	4	26.33	5	25.75
Japan	Asia	**18**	9,034	3	37,532	5	61.79	2	93.59	18	4.25	22	3.74	2	55.42	2	62.01
Cambodia	Asia	**19**	8,557	41	3,416	67	0.38	72	0.35	12	7.55	11	7.27	83	0.33	89	0.17
Thailand	Asia	**20**	8,385	33	4,957	13	15.89	12	12.40	15	5.10	13	6.54	41	1.82	52	0.60
Hong Kong	Asia	**21**	6,936	8	23,310	12	17.37	7	38.49	8	10.24	2	20.38	5	25.13	3	42.95
Mexico	America	**22**	6,821	19	10,664	29	2.67	30	3.75	6	11.19	3	15.06	24	3.67	33	1.98
Portugal	Europe	**23**	6,628	32	5,022	36	1.91	40	1.90	38	1.95	39	2.01	66	0.64	53	0.60
romania	Europe	**24**	5,923	31	5,261	46	1.28	47	1.25	36	2.10	34	2.50	69	0.56	49	0.72
Poland	Europe	**25**	5,534	18	11,029	47	1.19	42	1.58	31	2.41	37	2.18	19	5.30	23	3.19
Sri Lanka	Asia	**26**	5,532	57	2,380	43	1.39	63	0.62	16	4.84	20	4.48	96	0.20	97	0.14
Morocco	Africa	**27**	4,928	43	3,381	85	0.15	78	0.29	32	2.26	38	2.07	79	0.36	54	0.58
Australia	Oceania	**28**	4,534	21	9,147	7	51.23	6	44.94	54	0.90	53	0.93	10	12.57	12	10.25
Austria	Europe	**29**	4,328	22	9,040	22	6.16	22	4.61	64	0.70	52	0.97	23	3.88	21	3.84
Egypt	Africa	**30**	4,223	34	4,600	35	2.05	41	1.89	20	3.85	32	2.64	32	2.58	29	2.38
Tunisia	Africa	**31**	4,083	55	2,429	75	0.24	66	0.57	47	1.48	40	1.93	102	0.16	84	0.20
Czech Republic	Europe	**32**	3,855	30	5,547	34	2.06	34	2.60	52	1.13	46	1.24	38	2.01	41	1.64
Malaysia	Asia	**33**	3,612	36	4,138	23	5.96	19	6.41	30	2.62	30	2.70	28	2.87	31	2.26
Denmark	Europe	**34**	3,347	27	6,377	53	0.77	48	1.21	66	0.68	56	0.82	21	4.85	17	4.75
Honduras	America	**35**	3,245	69	1,525	81	0.20	91	0.15	19	4.05	17	4.98	103	0.16	113	0.09
Canada	America	**36**	2,979	16	14,009	33	2.49	23	4.36	23	3.46	16	5.41	9	12.58	8	14.13
Bulgaria	Europe	**37**	2,800	59	2,112	61	0.55	52	0.97	55	0.86	50	1.05	111	0.12	76	0.28
Switzerland	Europe	**38**	2,657	24	8,032	32	2.56	31	3.40	59	0.76	47	1.23	20	5.03	25	2.96
Brazil	America	**39**	2,568	25	7,379	16	9.73	28	3.90	21	3.61	36	2.24	22	4.58	42	1.13
Philippines	Asia	**40**	2,517	48	2,761	39	1.71	38	2.06	24	3.44	19	4.63	43	1.57	51	0.61
El Salvador	America	**41**	2,447	64	1,641	79	0.20	90	0.16	27	3.19	24	3.38	104	0.15	99	0.14
Sweden	Europe	**42**	2,010	29	5,984	59	0.58	50	1.10	71	0.44	73	0.41	26	3.36	26	2.90
Peru	America	**43**	1,879	60	2,069	51	0.91	58	0.87	40	1.90	42	1.86	47	1.40	64	0.43
Hungary	Europe	**44**	1,772	54	2,440	56	0.65	59	0.85	69	0.50	58	0.78	97	0.19	69	0.37
Slovakia	Europe	**45**	1,702	46	3,035	63	0.50	57	0.92	75	0.38	67	0.53	53	1.13	57	0.49
Greece	Europe	**46**	1,576	49	2,687	49	1.13	46	1.38	79	0.36	61	0.71	63	0.74	35	1.81
Lithuania	Europe	**47**	1,514	72	1,494	66	0.42	67	0.49	74	0.38	75	0.35	98	0.19	101	0.14
Myanmar	Asia	**48**	1,498	63	1,674	106	0.05	123	0.01	39	1.92	66	0.55	74	0.47	120	0.06
Guatemala	America	**49**	1,496	74	1,305	74	0.25	71	0.36	33	2.24	26	2.79	115	0.11	85	0.19
Nicaragua	America	**50**	1,484	100	515	118	0.02	127	0.01	37	1.98	62	0.69	133	0.07	118	0.07
