# Supplementary material for: Countries’ positions in the international global value networks: Centrality and economic performance
Source: Appl Netw Sci. 2017 Jul 12;2(1):21. doi: 10.1007/s41109-017-0041-4 (PMC6214273; doi:10.1007/s41109-017-0041-4)
Supplement: Supplementary file 1 — Appendix. (ZIP 258 kb) [file 41109_2017_41_MOESM1_ESM.zip › TableS1.pdf]

## Electronics

| | Continent | **Rank Exports 2014** | Exports ($M) 2014 | Rank Imports 2014 | Imports ($M) 2014 | Rank Upstreamness 2014 | Upstreamness 2014 | Rank Upstreamness 2007 | Upstreamness 2007 | Rank Midstreamness 2014 | Midstreamness 2014 | Rank Midstreamness 2007 | Midstreamness 2007 | Rank Downstreamness 2014 | Downstreamness 2014 | Rank Downstreamness 2007 | Downstreamness 2007 |
|---|---|---|---|---|---|---|---|---|---|---|---|---|---|---|---|---|---|
| China | Asia | **1** | 658,103 | 2 | 244,933 | 3 | 37.90 | 3 | 56.81 | 1 | 100.00 | 1 | 100.00 | 27 | 3.24 | 15 | 6.20 |
| Taiwan | Asia | **2** | 175,854 | 11 | 53,789 | 1 | 100.00 | 1 | 100.00 | 8 | 8.64 | 8 | 16.83 | 20 | 4.21 | 19 | 4.73 |
| South Korea | Asia | **3** | 148,891 | 8 | 72,569 | 2 | 59.50 | 4 | 53.00 | 5 | 13.47 | 6 | 21.00 | 10 | 9.30 | 10 | 8.87 |
| USA | America | **4** | 145,273 | 1 | 295,183 | 5 | 21.85 | 5 | 42.50 | 6 | 12.46 | 4 | 24.40 | 1 | 100.00 | 1 | 100.00 |
| Germany | Europe | **5** | 135,729 | 4 | 131,073 | 8 | 14.71 | 9 | 16.32 | 10 | 6.78 | 10 | 12.81 | 4 | 17.88 | 3 | 20.03 |
| Japan | Asia | **6** | 121,434 | 5 | 89,357 | 4 | 35.56 | 2 | 74.83 | 7 | 11.19 | 5 | 23.85 | 3 | 28.69 | 4 | 18.11 |
| Malaysia | Asia | **7** | 83,932 | 12 | 49,201 | 6 | 21.04 | 7 | 37.38 | 9 | 8.25 | 9 | 15.75 | 25 | 3.69 | 24 | 3.33 |
| Mexico | America | **8** | 80,214 | 7 | 79,529 | 11 | 6.15 | 10 | 11.32 | 3 | 18.71 | 3 | 31.97 | 5 | 12.04 | 11 | 8.58 |
| Singapore | Asia | **9** | 73,577 | 6 | 83,165 | 7 | 18.99 | 8 | 28.74 | 4 | 13.92 | 7 | 20.67 | 15 | 6.41 | 9 | 10.42 |
| Viet Nam | Asia | **10** | 44,400 | 16 | 32,672 | 12 | 5.77 | 33 | 0.97 | 11 | 5.71 | 32 | 1.24 | 23 | 4.01 | 32 | 2.06 |
| Netherlands | Europe | **11** | 43,022 | 13 | 48,997 | 21 | 1.12 | 19 | 2.63 | 17 | 2.22 | 14 | 5.41 | 6 | 11.40 | 5 | 15.27 |
| France | Europe | **12** | 40,221 | 10 | 56,555 | 13 | 2.82 | 13 | 5.94 | 18 | 2.13 | 16 | 4.15 | 7 | 10.67 | 7 | 11.40 |
| Philippines | Asia | **13** | 38,285 | 29 | 18,338 | 9 | 13.01 | 6 | 40.54 | 15 | 2.71 | 13 | 6.40 | 43 | 1.32 | 45 | 1.14 |
| Thailand | Asia | **14** | 37,327 | 15 | 33,939 | 10 | 6.54 | 11 | 10.95 | 12 | 5.16 | 11 | 8.27 | 16 | 6.32 | 18 | 4.78 |
| Italy | Europe | **15** | 30,280 | 17 | 32,272 | 19 | 1.28 | 16 | 3.53 | 24 | 1.29 | 20 | 2.69 | 18 | 4.32 | 16 | 5.83 |
| UK | Europe | **16** | 30,011 | 9 | 60,412 | 18 | 1.29 | 15 | 3.65 | 16 | 2.35 | 15 | 5.15 | 9 | 9.84 | 6 | 13.01 |
| Hong Kong | Asia | **17** | 26,593 | 3 | 232,208 | 14 | 2.80 | 12 | 10.46 | 2 | 21.02 | 2 | 39.78 | 2 | 53.95 | 2 | 45.25 |
| Czech Republic | Europe | **18** | 26,024 | 24 | 24,943 | 17 | 1.30 | 25 | 1.63 | 22 | 1.40 | 21 | 2.55 | 26 | 3.66 | 28 | 2.72 |
| Poland | Europe | **19** | 23,970 | 21 | 26,891 | 27 | 0.74 | 29 | 1.10 | 19 | 1.84 | 24 | 2.29 | 21 | 4.13 | 20 | 3.68 |
| Hungary | Europe | **20** | 20,214 | 26 | 19,675 | 25 | 0.91 | 24 | 1.67 | 23 | 1.34 | 17 | 3.94 | 40 | 1.51 | 22 | 3.58 |
| Austria | Europe | **21** | 17,128 | 31 | 16,970 | 20 | 1.14 | 22 | 1.82 | 25 | 0.96 | 29 | 1.40 | 32 | 2.21 | 33 | 1.74 |
| Sweden | Europe | **22** | 16,881 | 28 | 18,699 | 28 | 0.68 | 23 | 1.71 | 34 | 0.68 | 28 | 1.49 | 42 | 1.40 | 31 | 2.16 |
| Spain | Europe | **23** | 16,427 | 23 | 25,145 | 30 | 0.60 | 26 | 1.55 | 31 | 0.70 | 18 | 3.43 | 22 | 4.02 | 17 | 5.45 |
| Slovakia | Europe | **24** | 14,222 | 34 | 13,849 | 36 | 0.28 | 38 | 0.47 | 27 | 0.87 | 25 | 2.13 | 38 | 1.68 | 54 | 0.73 |
| Switzerland | Europe | **25** | 13,596 | 33 | 14,814 | 23 | 1.09 | 21 | 2.03 | 30 | 0.77 | 34 | 1.02 | 28 | 2.98 | 36 | 1.52 |
| Indonesia | Asia | **26** | 12,085 | 32 | 16,897 | 22 | 1.11 | 17 | 3.32 | 21 | 1.54 | 19 | 2.80 | 19 | 4.24 | 27 | 2.90 |
| Canada | America | **27** | 12,017 | 14 | 41,004 | 24 | 1.03 | 14 | 4.28 | 13 | 3.13 | 12 | 8.08 | 8 | 10.40 | 8 | 10.93 |
| Belg.- Lux. | Europe | **28** | 11,862 | 27 | 19,225 | 26 | 0.87 | 28 | 1.36 | 33 | 0.69 | 26 | 1.87 | 34 | 1.94 | 26 | 3.06 |
| romania | Europe | **29** | 11,424 | 36 | 11,033 | 32 | 0.54 | 39 | 0.46 | 37 | 0.48 | 44 | 0.33 | 62 | 0.56 | 53 | 0.73 |
| India | Asia | **30** | 10,411 | 19 | 29,226 | 29 | 0.63 | 35 | 0.89 | 20 | 1.72 | 23 | 2.38 | 11 | 9.06 | 12 | 8.30 |
| Turkey | Europe | **31** | 10,309 | 30 | 17,919 | 44 | 0.16 | 46 | 0.19 | 28 | 0.82 | 35 | 1.02 | 17 | 4.58 | 25 | 3.17 |
| Ireland | Europe | **32** | 9,613 | 46 | 5,477 | 40 | 0.20 | 18 | 2.65 | 40 | 0.41 | 33 | 1.11 | 61 | 0.59 | 46 | 1.11 |
| Costa Rica | America | **33** | 8,876 | 70 | 2,193 | 15 | 1.65 | 20 | 2.49 | 60 | 0.10 | 39 | 0.61 | 82 | 0.22 | 73 | 0.24 |
| Israel | Asia | **34** | 8,767 | 40 | 7,221 | 16 | 1.42 | 27 | 1.54 | 35 | 0.58 | 36 | 0.98 | 57 | 0.74 | 49 | 0.90 |
| Denmark | Europe | **35** | 8,322 | 38 | 9,101 | 35 | 0.29 | 36 | 0.71 | 38 | 0.45 | 30 | 1.30 | 58 | 0.73 | 43 | 1.19 |
| Finland | Europe | **36** | 7,504 | 42 | 6,745 | 33 | 0.45 | 34 | 0.92 | 41 | 0.40 | 22 | 2.38 | 50 | 0.94 | 23 | 3.42 |
| Portugal | Europe | **37** | 5,276 | 45 | 5,533 | 38 | 0.28 | 32 | 0.99 | 46 | 0.19 | 42 | 0.47 | 68 | 0.42 | 55 | 0.68 |
| Russian Federation | Europe | **38** | 4,646 | 18 | 31,827 | 47 | 0.11 | 42 | 0.31 | 26 | 0.91 | 37 | 0.86 | 12 | 8.41 | 13 | 7.39 |
| Morocco | Africa | **39** | 4,521 | 51 | 4,329 | 34 | 0.40 | 30 | 1.09 | 62 | 0.10 | 58 | 0.16 | 65 | 0.52 | 56 | 0.66 |
| Brazil | America | **40** | 4,324 | 22 | 26,845 | 39 | 0.23 | 37 | 0.47 | 14 | 2.81 | 27 | 1.84 | 24 | 3.94 | 30 | 2.38 |
| Estonia | Europe | **41** | 4,157 | 56 | 3,423 | 52 | 0.07 | 54 | 0.08 | 43 | 0.25 | 64 | 0.13 | 84 | 0.20 | 76 | 0.23 |
| Tunisia | Africa | **42** | 4,077 | 65 | 2,637 | 43 | 0.16 | 44 | 0.24 | 59 | 0.11 | 52 | 0.19 | 93 | 0.16 | 89 | 0.14 |
| Slovenia | Europe | **43** | 3,712 | 64 | 2,669 | 41 | 0.17 | 43 | 0.27 | 53 | 0.13 | 50 | 0.20 | 70 | 0.40 | 66 | 0.32 |
| Norway | Europe | **44** | 3,517 | 39 | 8,165 | 37 | 0.28 | 41 | 0.31 | 45 | 0.21 | 41 | 0.50 | 37 | 1.68 | 37 | 1.43 |
| United Arab Emirates | Asia | **45** | 3,329 | 20 | 28,735 | 50 | 0.08 | 55 | 0.08 | 32 | 0.70 | 38 | 0.81 | 14 | 6.43 | 21 | 3.60 |
| Ukraine | Europe | **46** | 2,709 | 53 | 4,025 | 53 | 0.06 | 51 | 0.10 | 54 | 0.13 | 59 | 0.15 | 54 | 0.79 | 47 | 1.09 |
| Bulgaria | Europe | **47** | 2,434 | 62 | 2,824 | 46 | 0.14 | 49 | 0.11 | 68 | 0.07 | 65 | 0.12 | 102 | 0.13 | 62 | 0.51 |
| Australia | Oceania | **48** | 2,414 | 25 | 21,101 | 42 | 0.17 | 40 | 0.44 | 29 | 0.78 | 31 | 1.27 | 13 | 7.40 | 14 | 6.88 |
| Egypt | Africa | **49** | 2,081 | 49 | 4,648 | 61 | 0.02 | 63 | 0.03 | 47 | 0.19 | 53 | 0.18 | 52 | 0.89 | 58 | 0.65 |
| South Africa | Africa | **50** | 2,037 | 37 | 9,717 | 54 | 0.04 | 50 | 0.11 | 42 | 0.33 | 43 | 0.45 | 30 | 2.76 | 29 | 2.50 |
